# Non-traumatic myositis ossificans mimicking a malignant neoplasm in an 83-year-old woman: a case report

**DOI:** 10.1186/1752-1947-4-270

**Published:** 2010-08-12

**Authors:** Jun Nishio, Kazuki Nabeshima, Hiroshi Iwasaki, Masatoshi Naito

**Affiliations:** 1 Department of Orthopaedic Surgery, Faculty of Medicine, Fukuoka University, Fukuoka, Japan; 2 Department of Pathology, Faculty of Medicine, Fukuoka University, Fukuoka, Japan

## Abstract

**Introduction:**

Myositis ossificans is a benign, self-limiting condition that usually affects young, athletically active men. To the best of our knowledge, this case report describes the oldest recorded patient with myositis ossificans.

**Case presentation:**

Our patient was an 83-year-old Japanese woman who presented with a one week history of a palpable mass in the left thigh. She had a history of surgery for transverse colon cancer and lung cancer at the ages of 73 and 80, respectively. Clinical and radiological examinations suggested a malignant neoplasm such as metastatic carcinoma or extraskeletal osteosarcoma. A diagnosis of myositis ossificans was made by core needle biopsy. Our patient was asymptomatic and had no recurrence at one year follow-up.

**Conclusion:**

Clinicians should consider myositis ossificans as a possible diagnosis for a soft tissue mass in the limb of an older patient, thereby avoiding unnecessarily aggressive therapy.

## Introduction

Myositis ossificans (MO) is a benign lesion of heterotopic ossification that chiefly affects active adolescents and young adults, with a slight male predominance. Any part of the body may be involved, but the anterior thigh is the most common site. This lesion is clearly related to trauma in 60% to 75% of cases [1]. Despite a clinically and histologically distinct entity, MO still causes considerable difficulties in diagnosis. We report a case of MO arising in the thigh of an older patient without any history of trauma.

## Case presentation

An 83-year-old Japanese woman was referred to our hospital with a one week history of a palpable mass in the anteriomedial aspect of the left thigh. There was no history of antecedent trauma, but our patient had a history of surgery for transverse colon cancer and lung cancer at the ages of 73 and 80, respectively. Physical examination revealed a tender, firm, and non-mobile mass that was 7 × 6 cm in size. Laboratory data were within the normal limits, including erythrocyte sedimentation rate, C-reactive protein and white blood cell counts.

**Figure 1:**
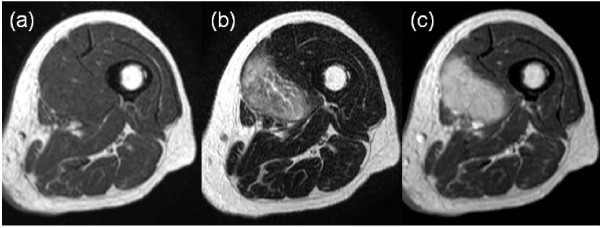
**A) MRI of the thigh showing iso signal intensity in a T1-weighted image**. B) Heterogeneous high signal intensity in a T2-weighted image. C) Diffuse enhancement in a post-contrast T1-weighted image.

**Figure 2:**
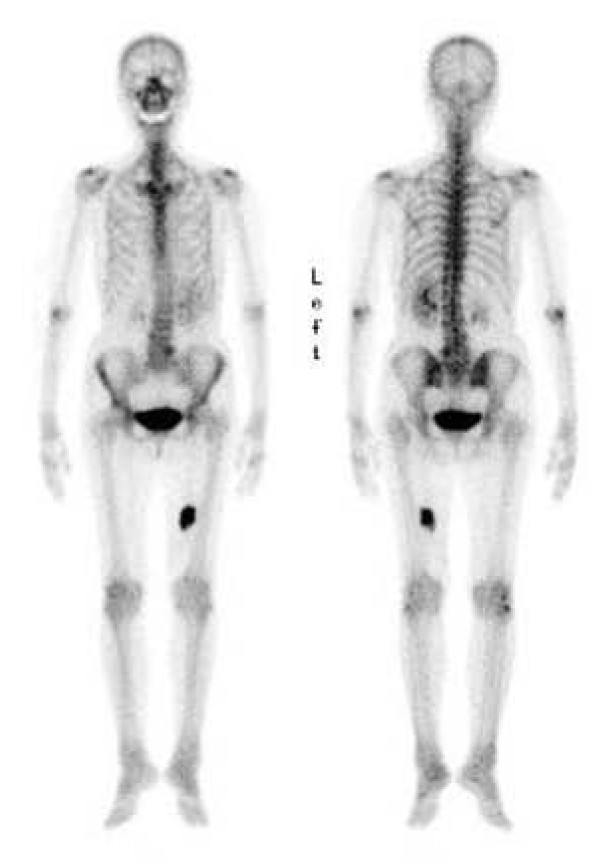
**Tc-99 m hydroxymethylenediphosphonate bone scintigraphy shows dense uptake in the medial soft tissue of the left thigh**.

A plain radiograph did not show any alteration. A magnetic resonance imaging (MRI) scan revealed a 6 × 5 cm poorly defined mass in the left vastus medialis muscle (Figure 1). On T1-weighted and T2-weighted images, the mass showed isointense and heterogeneous hyperintense signals, respectively. After intravenous gadolinium injection, the mass was enhanced significantly. Surrounding muscle edema was identified. Tc-99 m hydroxymethylenediphosphonate bone scintigraphy showed dense uptake in the medial soft tissue of the left thigh (Figure 2).

**Figure 3:**
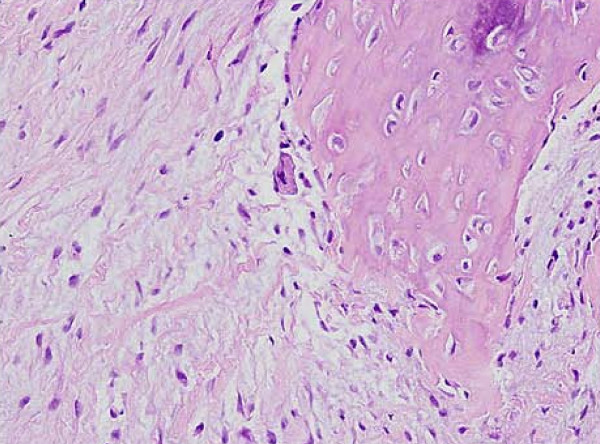
**Myositis ossificans composed of fibroblasts and focal deposits of osteoid**. Hematoxylin and eosin stain; original magnification ×100.

**Figure 4:**
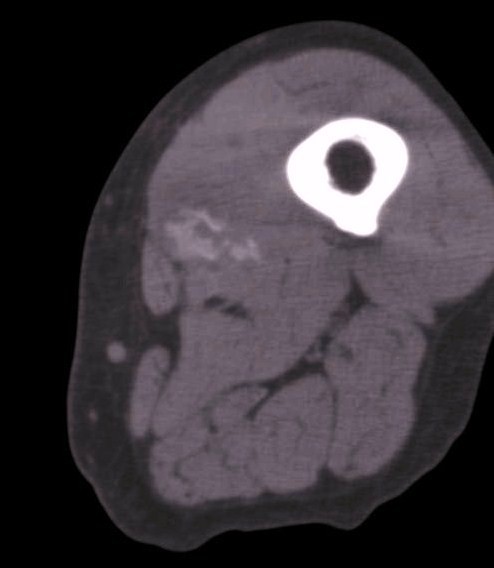
**CT of the thigh shows a lesion with peripheral ossification**.

The possibility of a malignant neoplasm was proposed, and a core needle biopsy was performed. Microscopically, the lesion was composed of a proliferation of fibroblasts admixed with foci of bone trabeculae lined by plump osteoblasts (Figure 3). Abnormal mitotic figures and nuclear pleomorphism were absent. These features were considered compatible with a diagnosis of MO. Our patient underwent a clinical and radiological follow-up. At three weeks after onset, a computed tomography (CT) scan demonstrated peripheral ossification of the lesion, thus further confirming MO (Figure 4). The symptoms resolved completely within two months. At one year follow-up, she was asymptomatic and had no recurrence.

## Discussion

MO, a benign condition, is commonly defined as a heterotopic ossification of soft tissues. MO can be seen at any age, but rarely occurs in babies or older patients [1]. To the best of our knowledge, the youngest documented patient was a five-month-old girl [2] and the oldest an 81-year-old woman [3].

The pathogenesis of MO is still uncertain. In cases with an apparent history of traumatic injury, it can be assumed that the process commences with tissue necrosis or hemorrhage followed by exuberant reparative fibroblastic and vascular proliferation with eventual ossification. In a small number of cases, etiologies may include burns, infections or drug abuse. However, non-traumatic cases have been documented in the literature [4,5]. In most of these cases, repetitive minor mechanical injuries, ischemia or inflammation have been implicated as possible causative factors [1]. Our case seems to belong to the non-traumatic MO group.

The zoning phenomenon of peripheral maturation is the most important diagnostic feature. Various radiological techniques have been applied for the detection and follow-up of MO [6]. Plain radiographs are usually normal at onset. In later stages, mineralization is present at the periphery and has a ring-like configuration. CT is the best imaging modality for diagnosing MO. MRI is a sensitive technique for identifying small, early lesions but is non-specific. Extensive muscle edema may be seen. Bone scintigraphy is very sensitive in the early detection of MO, demonstrating increased uptake in damaged muscle.

Differential diagnostic problems may arise in both early and late stages. In the earlier stages, the differential diagnoses should include extraskeletal osteosarcoma and synovial sarcoma when peripheral ossification is incomplete. In later stages, MO must be distinguished from parosteal or extraskeletal osteosarcoma and chondrosarcoma [6,7]. However, osteosarcoma usually lacks a zoning pattern of peripheral maturation.

The differential diagnosis may also include metastatic carcinoma in our case. Skeletal muscle metastasis is relatively rare. The most frequent affected sites include the abdominal wall, back, thigh, chest wall, and shoulder. The most common primary tumor is located in the lung and the most common histological diagnosis is adenocarcinoma [8-10]. Not surprisingly, ossifying skeletal muscle metastases have been reported in the literature [11,12]. In most cases, ossification is produced by osteoblasts originating by metaplasia from stromal fibroblasts. The clinical distinction between metastatic carcinoma to skeletal muscle and primary soft tissue tumor is critical because treatment and prognosis are markedly different. However, we were unable to eliminate the possibility of a metastatic carcinoma on the basis of clinical and radiological features.

The treatment of MO is usually conservative because of its self-limiting character and spontaneous regression. However, surgical excision is advised when joint function is impaired, neurovascular impingement is encountered, or the lesion is unusually large or painful. Surgery should only be undertaken on mature lesions.

## Conclusions

Although rare, MO should be considered in the differential diagnosis of older patients with a soft tissue mass. Without the characteristic radiological features, a biopsy is necessary to elucidate a diagnosis.

## Competing interests

The authors declare that they have no competing interests.

## Authors' contributions

JN managed our patient and drafted the manuscript. KN performed the histological examination of the specimen. KN, HI and MN participated in the design of the study and helped to draft the manuscript. All authors read and approved the final manuscript.

## Consent

Written informed consent was obtained from the patient for publication of this case report and any accompanying images. A copy of the written consent is available for review by the Editor-in-Chief of this journal.
